# Mapping the Intellectual Landscape of Giftedness in Early Childhood Through Comparative Topic Modeling

**DOI:** 10.3390/jintelligence14070119

**Published:** 2026-06-25

**Authors:** Simge Karakaş Mısır

**Affiliations:** Faculty of Education, Department of Special Education, Tokat Gaziosmanpaşa University, Tokat 60150, Türkiye; simge.karakasmisir@gop.edu.tr

**Keywords:** early childhood, gifted education, comparative topic modeling

## Abstract

The present study investigates the semantic structure, dominant themes, and temporal evolution of research on giftedness in early childhood through a comparative topic modeling approach. A final analytic sample (*n* = 518) of peer-reviewed journal articles indexed in the Scopus and Web of Science databases was analyzed. Three topic modeling methods, Latent Dirichlet Allocation (LDA), Structural Topic Modeling (STM), and BERTopic, were systematically compared using multiple evaluation metrics. BERTopic demonstrated the strongest overall performance, producing approximately 11% higher coherence than STM and approximately 34% higher coherence than LDA. In terms of diversity, it achieved 14% to 17% greater thematic variety and, according to the Gini coefficient, revealed a 58% to 60% more balanced thematic distribution. BERTopic-based analyses identified five major thematic axes: Socio-Linguistic Development and Family Context, Psychometric Intelligence, Identification, and Cognitive Differences, Program Access, Identification, and Educational Equity, Early Academic Skills and Cognitive Development, and Creativity, Higher-Order Thinking, and Enrichment Programs. Thematic mapping and topic similarity analysis were used to examine the semantic structure of the field, while linear regression-based trend analysis over the 1918–2026 publication period showed that family context, socio-linguistic development, and equity-related themes have gained increasing importance over time, whereas psychometric identification largely maintained its central position within the field. These findings indicate that the field is moving toward a more inclusive, semantically grounded, and equity-oriented perspective. However, they should be interpreted in light of the study’s reliance on article abstracts, the sensitivity of BERTopic clustering parameters, and the use of linear trend modeling.

## 1. Introduction

Giftedness in early childhood is difficult to define and study because advanced potential often emerges asynchronously across cognitive, social, emotional, and contextual domains. Giftedness refers to advanced potential or performance across cognitive, creative, socio-emotional, and domain-specific areas, rather than a status defined only by IQ or formal identification (Renzulli, 2012; Sternberg, 2024). This complexity is especially salient in young children, whose abilities may appear through asynchronous development, rapid learning, advanced language, play, curiosity, or context-dependent forms of performance (Gagné, 2015; Silverman, 2021; VanTassel-Baska & Wood, 2010). Therefore, research on giftedness in early childhood is characterized by conceptual diversity, methodological differences, and definitions that vary across studies (Bildiren, 2018; Brighton & Jarvis, 2017; Coates et al., 2009; Grant & Morrissey, 2019; Kauffman et al., 2024).

This conceptual and operational variability is particularly visible in identification processes. It has long been recognized that psychometric measures used with young children are controversial in terms of both reliability and cultural validity. Differences in identification criteria, test types, and cutoff thresholds generate substantial variability across studies, demonstrating that indicators of giftedness in early childhood cannot be reduced to a standardized profile (Carman, 2013; Peters et al., 2023). In this context, although the combined use of multiple data sources such as parent reports, teacher observations, and performance-based tasks has been recommended, no common assessment framework has yet been established in practice. International findings indicate that identification in early childhood settings is often shaped by teacher experience and institutional differences. The inadequacy of restricting early identification to criteria based solely on cognitive performance has made the need for context-sensitive and dynamic assessment models grounded in multiple data sources increasingly visible (Brighton & Jarvis, 2017; Pfeiffer, 2015).

Another dimension directly related to identification debates is socio-emotional development. In young gifted children, cognitive acceleration can often be accompanied by characteristics such as emotional intensity, heightened sensitivity, difficulty navigating social situations that require flexibility, and challenges in peer relationships (Sternberg, 2024). Studies conducted from the parental perspective indicate that families seek systematic support not so much for the child’s cognitive acceleration as for areas such as intense emotionality, perfectionistic tendencies, and asynchrony in peer relationships (Papadopoulos, 2021). Research based on family interviews shows that strong early cognitive indicators should be evaluated together with a distinctive emotional profile and demonstrates that emotional sensitivity and inner intensity, in particular, can directly affect adjustment to educational settings (De Souza Fleith et al., 2024). Qualitative findings based on caregiver interviews further confirm that socio-emotional complexity constitutes a primary concern for parents and that the search for systematic support is especially prominent in relation to being misunderstood in school contexts, matching with peers, and meeting emotional needs (Guilbault & Johnson, 2026).

Taken together, these findings point to a clear research problem: research on giftedness in early childhood has expanded, but its thematic organization remains fragmented. Prior field-level studies point to an uneven conceptual structure in gifted education research, with identification, cognitive performance, curriculum, and assessment receiving greater visibility (Cristina et al., 2025; Hernández-Torrano & Kuzhabekova, 2020; Şakar & Tan, 2025). At the same time, issues related to socio-emotional development, contextual conditions, equity, and inclusive educational access have been highlighted as areas requiring more sustained attention, particularly in early childhood and underrepresented populations (Mossberg et al., 2024; Worley & Young, 2023).

Against this background, the present study approaches thematic change not only as a matter of word-frequency patterns, but also as a semantic structure that can be examined through transformer-based representations. By comparing traditional topic modeling approaches with a semantic representation-based approach, the study combines quantitative model evaluation with qualitatively interpretable thematic mapping. This comparative design provides a more transparent basis for examining how themes in early childhood giftedness research are organized, how they change over time, and which conceptual areas become more or less visible across different modeling approaches (Abdelrazek et al., 2023; Khodeir & Elghannam, 2025).

### 1.1. Computational Topic Modeling: Statistical and Semantic Approaches

In educational sciences, literature synthesis is increasingly oriented toward statistical and semantic topic modeling techniques in response to the need for scalability and objectivity (Ahadi et al., 2022). These techniques assume different yet complementary roles in understanding the structural dynamics of the field.

Among statistical approaches, Latent Dirichlet Allocation (LDA) identifies dominant themes based on word co-occurrence patterns, whereas Structural Topic Modeling (STM) enables the analysis of the relationships between themes and variables such as publication year, journal, or discipline by incorporating metadata into the model structure (Blei et al., 2003; Roberts et al., 2014). However, the short and context-sensitive nature of educational data may cause thematic boundaries to become blurred in models based solely on word frequency. Among semantic approaches, BERTopic analyzes texts through deep semantic embedding representations by using transformer-based language models (Galli et al., 2024). This architecture goes beyond statistical matches at the word level to capture the conceptual core of the text and produces more coherent and differentiated thematic structures, particularly in heterogeneous disciplines where similar words may carry different meanings across contexts (Abdelrazek et al., 2023; Khodeir & Elghannam, 2025). These features make BERTopic a strong candidate for mapping the thematic architecture of a conceptually multilayered field such as giftedness in early childhood.

The methodological challenge, therefore, is to examine a fragmented and conceptually heterogeneous literature in a way that is systematic, reproducible, and sensitive to semantic variation. This is directly aligned with the research questions of the study, which require identifying dominant themes, tracing thematic trends over time, and detecting comparatively underrepresented conceptual areas across the full bibliographic corpus. These aims cannot be addressed adequately through a purely descriptive synthesis, because related concepts may appear under different labels and similar terms may carry different meanings across developmental, educational, psychological, and policy contexts. Accordingly, topic modeling is necessary not only for summarizing a large body of literature, but also for revealing its latent thematic organization and temporal movement. Comparing frequency-based topic modeling approaches with semantic representation-based approaches further strengthens this rationale by showing how different models represent the same literature and how transformer-based representations may offer a more conceptually sensitive mapping of early childhood giftedness research (Abdelrazek et al., 2023; Ahadi et al., 2022; Şakar & Tan, 2025).

### 1.2. Purpose and Contribution of the Study

The central aim of this study is to examine the intellectual structure, thematic patterns, and temporal transformation of the literature on giftedness in early childhood within the framework of a comparative topic modeling approach. In this regard, the study goes beyond merely providing a descriptive mapping of the literature and systematically compares how the same dataset is structured by three different topic modeling algorithms, namely LDA, STM, and BERTopic. This comparison is conducted using multidimensional performance indicators, including topic coherence, diversity, distributional balance, and cluster separation. In doing so, the study seeks to analytically demonstrate how methodological choices are reflected in the conceptual representation of the literature.

This study contributes to research in this field on three main levels. First, from a methodological perspective, it is one of the limited number of studies to comparatively examine probabilistic and transformer-based topic modeling approaches on the same dataset in the context of giftedness in early childhood. In this respect, the study not only reveals the thematic structure of the research literature but also systematically evaluates how different algorithm-based approaches represent this body of scholarship.

Second, the study moves beyond producing a static thematic map by examining the temporal evolution of the field through linear regression-based trend analysis. In this way, it identifies which themes have demonstrated continuity, which have shown an upward trajectory, and which have relatively weakened in research on giftedness in early childhood.

Third, the study offers an original contribution at the theoretical, practice-oriented, and policy-focused levels. In particular, the representational capacity provided by semantic embedding-based models makes research axes centered on equity, family context, inclusivity, and policy more visible than is possible through traditional frequency-based approaches. In this respect, the study contributes to the reconsideration of the research agenda on giftedness in early childhood within a more inclusive, multidimensional, and policy-sensitive framework.

Accordingly, the study seeks to answer the following research questions:

RQ1: What dominant thematic structures are present in the literature on giftedness in early childhood?

RQ2: How does the BERTopic model, in comparison with the other models, reveal thematic trends in the literature and changes in these trends over time?

RQ3: Which conceptual gaps and relatively underrepresented areas of research stand out in the literature?

RQ4: To which themes do the current thematic trends indicate future research priorities are likely to converge?

## 2. Methods

This section describes the comparative topic modeling design used to examine thematic structures and temporal trends in the literature on giftedness in early childhood. LDA, STM, and BERTopic were comparatively evaluated for topic discovery and trend analysis. In the methodology section, within the scope of trend analysis, the traditional word frequency-oriented and statistically grounded STM and LDA models, together with semantic inference-oriented BERT topic modeling, are comparatively addressed in terms of topic discovery and trend analysis.

### 2.1. Research Design

**Figure 1:**
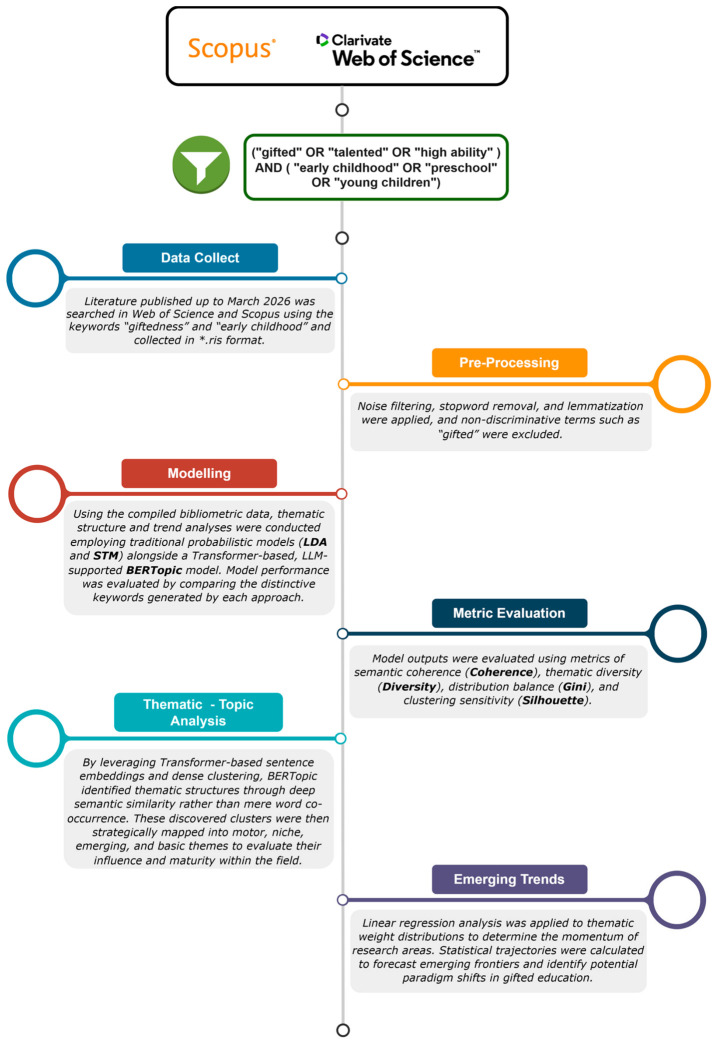
Overview of the Research Process.

This study is built on a multi-stage research design developed to reveal the conceptual structure, thematic diversity, and temporal evolution of the academic literature on giftedness in early childhood through a systematic and data-driven approach. The research process consists of five interrelated and sequential stages: (i) data collection, (ii) preprocessing, (iii) topic modeling, (iv) metric-based model evaluation, and (v) thematic and temporal trend analysis. Figure 1 presents the conceptual framework and research design of the study.

Web of Science and Scopus were used jointly to ensure coverage of interdisciplinary scholarship and high-impact publications. The data search strategy for topic-based trend analysis was structured to directly associate concepts of giftedness with the context of early childhood. The retrieved data were subjected to preprocessing to make them suitable for analytical modeling. In this process, semantically non-distinctive and noise-generating words were removed, and coherent multi-word expressions were employed to strengthen sentence-level integrity. In addition, removing high-frequency general terms that were relevant to the topic but had limited discriminative power enhanced differentiation across themes. During the modeling stage, LDA, STM, and BERTopic were applied comparatively rather than merely in parallel. The rationale for using these three approaches was that they represent complementary topic-modeling paradigms. LDA was used as a probabilistic word co-occurrence baseline, STM was included as a metadata-sensitive extension of probabilistic topic modeling, and BERTopic represented a transformer-based semantic embedding approach. This design allowed the same corpus to be evaluated through frequency-based, metadata-informed, and semantic representation-based assumptions. Model outputs were then compared in terms of dominant keywords, thematic structures, and predefined evaluation metrics, including topic coherence, diversity, topic distribution balance, and clustering sensitivity. The selected model was used to examine how thematic structures changed over time. Linear trend analyses identified research foci that increased, remained stable, or declined. Statistical inferences were made to interpret the emerging research directions.

### 2.2. Data Sources and Research Strategy

The dataset for this study was obtained from the Scopus and Web of Science (WoS) databases, which are widely used internationally in the fields of education and psychology. The data were collected as of March 2026. Both databases were selected because of their capacity to represent academic production in the field of giftedness in early childhood in a comprehensive and systematic manner. This integrated search strategy was designed to capture both the quantitative breadth of the field and publications of high academic quality.

**Figure 2:**
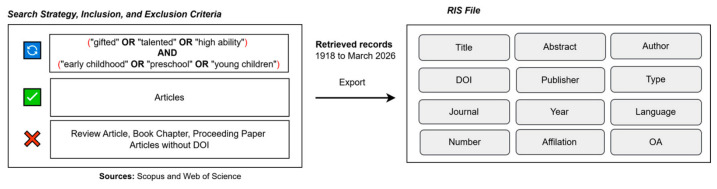
Search Strategy, Inclusion and Exclusion Criteria.

The retrieved records were screened according to the inclusion and exclusion criteria shown in Figure 2 and were exported in standard RIS file format. In Figure 2, the green check mark indicates the inclusion criteria (journal articles), the red cross represents the exclusion criteria (review articles, book chapters, conference papers, and publications without a DOI), and the blue search icon denotes the search query used to retrieve records from the databases. To improve transparency and replicability, the database-specific search procedures are reported in detail in this section. The searches were conducted in Scopus and Web of Science in March 2026. No topic-specific lower publication-year limit was set during the search. The final retrieved records covered the period from 1918 to March 2026. The search was limited to the title and abstract fields in order to identify publications directly addressing giftedness in early childhood.

In Scopus, the search was conducted using the TITLE-ABS field code with the following query.

TITLE-ABS ((“gifted” OR “talented” OR “high ability”) AND (“early childhood” OR “preschool” OR “young children”)) AND DOCTYPE(ar) AND SRCTYPE(j) AND PUBYEAR < 2027.

In Web of Science, the search was conducted using the title (TI) and abstract (AB) field codes with the following query.

((TI = (“gifted” OR “talented” OR “high ability”) OR AB = (“gifted” OR “talented” OR “high ability”)) AND (TI = (“early childhood” OR “preschool” OR “young children”) OR AB = (“early childhood” OR “preschool” OR “young children”))) AND DT = (Article) AND PY = (1900–2026).

Boolean operators were used to combine synonymous terms with OR and to connect the giftedness-related and early-childhood-related concept blocks with AND. No database-level language restriction was applied during retrieval. After export, only journal articles with DOI information, complete bibliographic metadata, and usable title and abstract text were retained for analysis. Review articles, book chapters, conference proceedings, duplicate records, and records without complete abstract information were excluded. As shown in Table 1, a total of 745 records were initially retrieved from Scopus and Web of Science. Following validation procedures and duplicate removal, 518 unique journal articles were retained for the final dataset.

### 2.3. Text Preprocessing

The primary aim of the text preprocessing stage was to reduce linguistic noise with limited semantic contribution to the analysis and to ensure that the topic modeling process focused on distinctive conceptual structures. Accordingly, the dataset was subjected to a systematic process of cleaning and normalization. First, stopwords consisting of conjunctions, prepositions, and frequently used functional words were removed from the text (Sánchez-Rada & Iglesias, 2019). This step was intended to prevent words with very high frequency but limited substantive content from being treated by the model as thematically meaningful.

Subsequently, the words were subjected to lemmatization. Lemmatization provides normalization by relying not on the surface forms of words but on their grammatically meaningful dictionary forms (Ozturkmenoglu & Alpkocak, 2012). This approach prevented words that represented the same concept but differed formally because of inflections or derivational variations from being interpreted by the model as separate themes.

To preserve textual context and strengthen conceptual integrity, word groups that generated meaning together were taken into account during the analysis process. In cases where individual words offered limited semantic depth, co-occurring expressions were assumed to provide a higher level of conceptual specificity. Accordingly, words that were frequently encountered in the examined subject area but had limited discriminative value when considered in isolation were evaluated within their contextual co-occurrences, thereby enhancing the sensitivity of the thematic analysis. In addition, certain repetitive expressions with limited semantic contribution across the text and a weakening effect on thematic differentiation were deliberately removed. In the final stage, meaningful word combinations were constructed in order to preserve the contextual integrity of concepts that could not be adequately represented through isolated usage.

### 2.4. Analytical Models

This section addresses the theoretical foundations of the LDA, STM, and BERTopic methods. While LDA and STM adopt topic extraction approaches based on word frequencies and statistical assumptions, BERTopic performs topic extraction by using transformer-based semantic representations. In this respect, BERTopic offers an approach that takes semantic similarities into account more effectively.

#### 2.4.1. BERTopic

**Figure 3:**
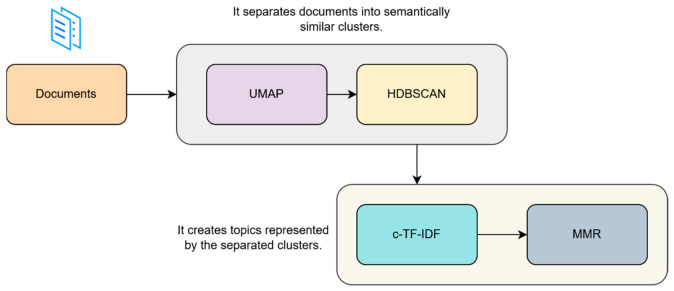
BERTopic Model.

Unlike traditional probabilistic topic modeling approaches, BERTopic constructs the semantic representations of documents through contextual text embeddings and integrates these representations with density-based clustering methods (Galli et al., 2024). This approach makes a substantial contribution to generating more coherent, distinguishable, and interpretable thematic structures, particularly in disciplines such as educational sciences, where conceptual cohesion is relatively weak and terminological diversity remains pronounced. A schematic representation of the BERTopic workflow is provided in Figure 3.

BERTopic is a topic modeling approach that analyzes texts not only according to word frequencies but also by taking semantic relationships into account. In this method, documents are transformed into numerical embedding vectors through pre-trained transformer-based language models such as BERT, RoBERTa, and Sentence-BERT. In this way, the contextual meaning of texts is projected into vector space. The resulting high-dimensional vectors are then reduced to a lower-dimensional structure more suitable for clustering by using Uniform Manifold Approximation and Projection (UMAP). Subsequently, similar documents are automatically clustered with the help of the Hierarchical Density-Based Spatial Clustering of Applications with Noise (HDBSCAN) algorithm, while documents characterized as noise are separated. In the final stage, class-based TF-IDF (c-TF-IDF) is applied to identify distinctive key terms for each cluster. Through this integrated structure, BERTopic makes it possible to generate interpretable and semantically coherent topic representations from large and conceptually heterogeneous text collections (Uğuz & Tülü, 2024).

#### 2.4.2. Latent Dirichlet Allocation (LDA)

Latent Dirichlet Allocation (LDA) emerged as a generative probabilistic method used to model discrete data collections such as text corpora (Blei et al., 2003). Rather than treating a document as belonging to a single topic, LDA represents it as a mixture of multiple topics. It first determines the proportions of topics present in each document. Then, each word in the document is associated with a topic according to these topic proportions. In this way, a text is not assigned to only one topic but can contain multiple topics with different weights. This makes it possible to model the multidimensional structure of the text more realistically.

Owing to its flexible and multidimensional structure, LDA provides a powerful tool for discovering latent themes within texts in large-scale data analyses. The model defines each extracted topic as a statistical mixture of words that frequently co-occur within that topic. It has been widely and effectively used, particularly in comprehensive literature reviews and bibliometric research, to make sense of large text collections, group latent subthemes, and chronologically map the evolution of these topics over time (Cristina et al., 2025). In addition to serving as an effective tool for identifying dominant topics, determining trends, and delineating the scope and subthemes of a particular topic in the literature, LDA has also proved useful for inferring future trends.

#### 2.4.3. Structural Topic Modeling (STM)

Structural Topic Modeling (STM) is a machine learning algorithm used to uncover latent topics, themes, and trends within a given body of text (Roberts et al., 2019). By analyzing the correlations among topics derived from texts, STM is able to identify relationships between topics. It also enables the exploration of themes, latent structures, and temporal trends within large text datasets by integrating metadata into the model (Chen et al., 2026).

In the context of educational research, the use of STM offers important methodological advantages compared with traditional systematic literature review and manual content analysis (Şakar & Tan, 2025). First, STM draws on high computational power to analyze the continuously expanding body of academic publications and can examine large datasets rapidly, scalably, and holistically. Because it does not rely on predefined coding schemes, it reduces researcher bias. Whereas the theoretical orientations and prior assumptions of coders may influence the process in manual content analysis, STM adopts a data-driven approach. In this way, complex and latent thematic structures that are difficult to detect through human review become more visible. It also allows for a deeper analysis of the conceptual boundaries and developmental dynamics of the field and generates strong inferences regarding future research directions.

STM is an approach that extends the topic modeling framework of Latent Dirichlet Allocation (LDA) by incorporating contextual variables into the analysis. LDA estimates latent topic distributions in documents solely on the basis of textual content and assumes that topics are independent of one another (Jiang et al., 2025). By contrast, STM integrates document-level metadata, such as publication year, country, or research design, into the model, making it possible to analyze how topic prevalence and topic content vary according to contextual factors. In this respect, STM reveals not only which topics exist, but also under which conditions and in what ways these topics differ.

### 2.5. Metrics

A robust multidimensional and model-neutral evaluation framework incorporating four complementary metrics was established before model comparison in order to evaluate LDA, STM, and BERTopic on a common analytical basis. These metrics were topic coherence, diversity, the Gini coefficient, and the silhouette score (Newman et al., 2010; O’Callaghan et al., 2015). They were selected because, when evaluated together, they measure the semantic, structural, distributional, and clustering-related properties of the extracted themes from different perspectives. This framework therefore allowed a comprehensive comparison between probabilistic and transformer-based topic modeling approaches. The final model was selected according to predefined decision rules. Higher topic coherence was treated as the primary criterion. Higher diversity, a lower Gini coefficient for balanced thematic representation, and a positive or comparatively stronger silhouette score were used as complementary criteria. No single metric was considered sufficient for model selection. Each metric yields values within specific numerical ranges, and these ranges informed the interpretation of model performance.

Topic coherence measures the conceptual integrity within a topic on the basis of the co-occurrence patterns and semantic proximity of the highest-probability words representing that topic in the text. In this study, topic coherence was calculated using the $C_{v}$ measure (Röder et al., 2015). Values generally range from 0 to 1, with higher scores indicating that the keywords within a topic form a more coherent and meaningful conceptual structure. In the context of educational research, this metric is important for assessing whether the model generates themes that are not only statistically valid but also theoretically interpretable. However, because topic coherence is sensitive to the frequency with which words co-occur in the text, superficially related but frequently co-occurring concepts may also produce high scores. For this reason, it is not a sufficient indicator of quality on its own.

Diversity is calculated as the ratio of unique words appearing in the top-word lists of different topics to the total number of words (Chagnon et al., 2024). In this study, diversity was calculated as the proportion of unique words across the combined top words of all topics. Values range from 0 to 1, where values close to 0 indicate high word repetition and thematic overlap, whereas values close to 1 indicate low overlap between topics and a more differentiated thematic structure. However, excessively high diversity values may suggest that the topics are overly fragmented and that internal coherence may be weakened. For this reason, diversity is more informative when evaluated together with topic coherence (O’Callaghan et al., 2015).

The Gini coefficient was used to measure the balance of topic distribution within the dataset and takes values between 0 and 1 (Petersson, 2023). In this study, the Gini coefficient was calculated on the basis of topic proportions within the dataset. A value of 0 indicates a balanced distribution in which all topics are equally represented, whereas a value of 1 indicates a concentrated structure in which a single topic is dominant. In the context of topic modeling, a low Gini coefficient indicates that thematic diversity is more evenly distributed, whereas a high value indicates that certain themes dominate the literature. However, the Gini coefficient is not a normative indicator of “good” or “bad.” If the literature is inherently concentrated around particular themes, a high Gini coefficient may reflect the actual intellectual structure. Therefore, the Gini coefficient is an explanatory indicator that describes structural concentration rather than performance.

The silhouette score is a clustering validity index that measures how well documents are separated according to the clusters to which they are assigned in the embedding space, and it ranges from −1 to +1 (Yan & Zhang, 2024). In this study, the silhouette score was calculated using cosine distance. Values approaching +1 indicate that documents are more similar within their own clusters and more distant from other clusters, suggesting that the clusters possess both internal coherence and clear separation. Values close to 0 indicate that boundaries between clusters overlap or that separation is weak, whereas negative values suggest that documents may be closer to an alternative cluster, indicating possible misclassification. In this study, the silhouette score was used as a complementary indicator for evaluating relative cluster separation, particularly in relation to transformer-based semantic representations. Because silhouette values can be affected by similarity measures in high-dimensional embedding spaces, the clustering algorithm used, and the number of clusters, this metric was interpreted together with coherence, diversity, and the Gini coefficient rather than as an independent basis for model selection.

Taken together, topic coherence, diversity, the Gini coefficient, and the silhouette score provide a holistic performance framework. Topic coherence assesses semantic interpretability. Diversity assesses conceptual differentiation. The Gini coefficient describes structural concentration. The silhouette score evaluates cluster separation in the embedding space. Because model selection based on a single metric may be methodologically misleading, this study adopted a multi-criteria evaluation approach and systematically compared the models across semantic, structural, and distributional dimensions.

## 3. Results

### 3.1. Temporal and Conceptual Mapping of the Literature

Before examining the thematic structure of the literature on giftedness in early childhood, the temporal development and conceptual organization of the field were addressed at a macro level. Temporal analysis reveals trends and shifts in publication output, whereas conceptual mapping shows which concepts are central and which remain peripheral or emerging themes. Considered together, these two analyses indicate that the literature has not only expanded quantitatively but has also diversified conceptually. This framework provides an essential context for the subsequent topic modeling analyses.

**Figure 4:**
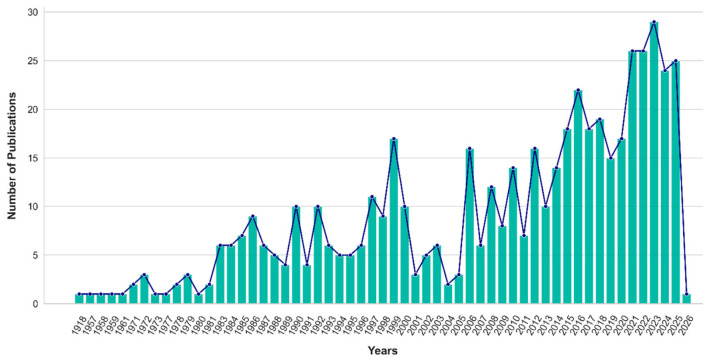
Annual Scientific Output: An upward trend until 2026.

Figure 4 presents the changes in the annual number of publications in the field of giftedness in early childhood up to 2026. The findings indicate a marked upward trend after 2016. In particular, research output appears to have accelerated after 2018 and reached its peak during the 2021–2024 period.

This increase suggests that the field has evolved beyond being a research area centered solely on identification and has developed into an interdisciplinary domain encompassing broader developmental and pedagogical dimensions. The low value for 2026 is attributable to the fact that the year has not yet been completed.

**Figure 5:**
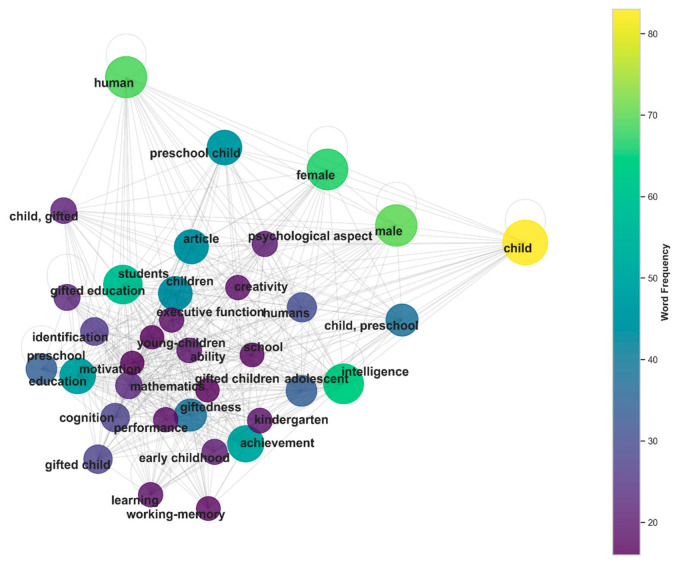
Keyword Network.

The Keyword Network shown in Figure 5 reveals the multilayered and fragmented nature of the conceptual structure in the literature, thereby clearly demonstrating the methodological need for a more advanced semantic modeling approach. The findings derived from the network indicate that core concepts such as “child,” “students,” “children,” and “gifted education” are positioned at the center of the network with high co-occurrence frequencies. In contrast, traditional focal areas such as “identification,” “intelligence,” and “assessment” display dense connection patterns within the core region, whereas themes such as “creativity,” “motivation,” “social development,” and “working memory” are located more peripherally, forming lower-density and relatively specialized clusters (in purple and dark blue tones).

### 3.2. Comparison of Analytical Methods (Why BERTopic?)

The LDA, STM, and BERTopic models were compared using four complementary metrics: topic coherence, diversity, distributional balance as measured by the Gini coefficient, and clustering separation quality as measured by the silhouette score. The aim was not only to assess statistical performance but also to determine the extent to which these models could meaningfully and distinctly represent the conceptual structure of the literature.

Table 2 presents a comparative overview of the dominant keywords extracted by the LDA, STM, and BERTopic models. For each model, five themes are listed according to their relative prominence, thereby illustrating how the same body of literature is represented through different analytical approaches.

The findings show that the LDA and STM models are built on probabilistic structures based on word frequency and therefore identify themes according to patterns of word co-occurrence. Although this approach is functional in generating broad thematic clusters, it may in some cases lead to conceptual boundaries that are more flexible or overlapping.

By contrast, the BERTopic model takes into account the contextual and semantic relationships between words through transformer-based embedding representations and structures themes within a more integrated semantic framework. In particular, the clearer and more conceptually coherent differentiation of themes such as identification, cognitive differences, and creativity in BERTopic indicates that the model possesses a semantic inference capacity that extends beyond simple word co-occurrence.

Table 3 compares the performance of the LDA, STM, and BERTopic models within a multi-criteria framework that jointly considers semantic, structural, and distributional dimensions. An examination of the topic coherence values shows that BERTopic (0.3958) provides approximately 11% greater within-topic conceptual coherence than STM (0.3554) and approximately 34% greater coherence than LDA (0.2958). In terms of diversity, BERTopic (0.8200) also produces values approximately 17% higher than STM and 14% higher than LDA, indicating lower overlap across topics and more distinct thematic differentiation.

The low Gini coefficient value (0.0396) indicates that the themes are distributed more evenly across the dataset. This value points to a thematic structure that is approximately 60% more balanced than STM and 58% more balanced than LDA.

With regard to the silhouette score, BERTopic produces a positive value (0.0468), whereas STM (−0.0236) and LDA (−0.0461) yield negative values. This finding suggests that BERTopic is able to separate documents more distinctly in the embedding space, while the other two models may exhibit greater overlap between clusters.

By achieving improvements of up to 34% in topic coherence, 17% in diversity, and 60% in distributional balance, BERTopic demonstrates a stronger and more balanced performance than the other models. This finding indicates that the model is methodologically more advantageous in terms of thematic structuring and differentiation.

**Figure 6:**
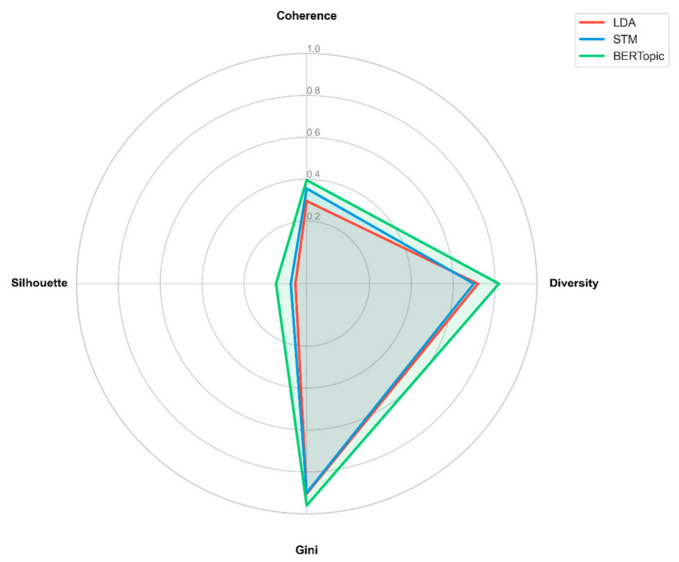
Model Performance Comparison.

Figure 6 presents a radar chart based on the data in Table 3, visually comparing the relative positions of the models across the four performance metrics. The chart shows that the BERTopic area forms a broader surface than those of the other models. In particular, its highest values on the coherence and diversity axes indicate that it provides superior performance in terms of within-topic conceptual coherence and between-topic differentiation.

When these metric results are considered together, BERTopic is understood to exhibit a more balanced and robust performance in terms of topic coherence, diversity, the level of structural concentration reflected by the Gini coefficient, and separation performance in the embedding space as indicated by the silhouette score. Evaluated jointly, these multidimensional performance metrics show that the BERTopic model offers not only statistical superiority but also a clear methodological advantage in terms of semantic differentiation and thematic structuring. This result provides a methodological justification for selecting BERTopic in the subsequent analyses of thematic mapping and trend projection.

Table 4 further substantiates why the BERTopic model, whose methodological superiority was established through multidimensional performance metrics, was preferred for thematic mapping and trend projection analyses. Demonstrating a more coherent and differentiated thematic structure through topic coherence, diversity, and especially its positive silhouette score, BERTopic was adopted as the main analytical model because of its capacity to structure the intellectual patterns of the literature through semantically informed representations.

Accordingly, Table 4 presents the five main topics extracted by BERTopic together with their theoretically assigned thematic counterparts. The resulting keyword clusters reflect both established and emerging academic themes in the literature, including socio-linguistic and family context, psychometric intelligence and identification, program access and educational equity, early academic and cognitive development, and creativity and higher-order thinking. This finding indicates that BERTopic provides not only topic extraction but also meaningful and interpretable thematic structuring. The themes were determined through expert evaluation.

### 3.3. BERTopic Thematic Areas and Trend Analysis

The thematic structure and temporal trends of the literature on giftedness in early childhood were examined through the multidimensional visual and analytical outputs generated by the BERTopic model. The characteristic keywords and thematic distributions associated with the themes identified by the model were evaluated, and topic-based word cloud visualizations were analyzed to reveal the conceptual density of the topics. The structural positioning and relative importance of the themes were examined through a thematic map (strategic diagram). In addition, the degree of semantic proximity and differentiation among the themes was assessed using a topic similarity heatmap. To identify the themes that are most prominent in the literature, topic distribution ratios were examined, while changes in conceptual orientations over time were revealed through thematic trend analyses. Taken together, these analyses aim to explain the conceptual structure of research on giftedness in early childhood and its developmental trajectory over time within a holistic framework.

**Figure 7:**
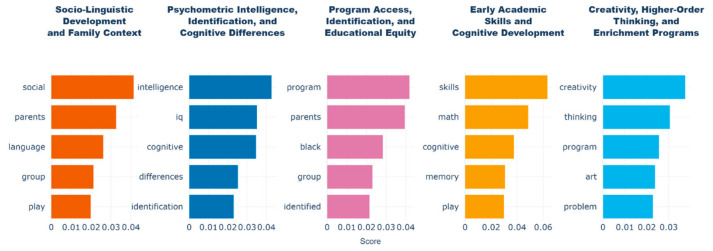
Keywords and Thematic Distribution Obtained with the BERTopic Model.

Figure 7 presents the characteristic keywords and thematic intensities of the themes identified by the BERTopic model. The results indicate that the literature on giftedness in early childhood is concentrated around five main thematic axes.

The first theme, Socio-Linguistic Development and Family Context, highlights concepts such as social interaction, parental involvement, and language development. The second theme, Psychometric Intelligence, Identification, and Cognitive Differences, focuses on the identification of intelligence, IQ, and cognitive differences. The third theme, Program Access, Identification, and Educational Equity, reflects discussions concerning access to educational programs, identification processes, and equity in education.

The fourth theme, Early Academic Skills and Cognitive Development, represents early academic competencies such as mathematics, cognitive skills, and memory. The fifth theme, Creativity, Higher-Order Thinking, and Enrichment Programs, encompasses research on creativity, higher-order thinking skills, and enrichment programs.

The resulting thematic structure demonstrates that, through BERTopic’s transformer-based semantic embedding approach, concepts in the literature can be grouped not only according to word frequencies but also on the basis of meaning relations. This reveals the multidimensional nature of research on giftedness in early childhood in the form of more coherent and interpretable thematic clusters.

**Figure 8:**
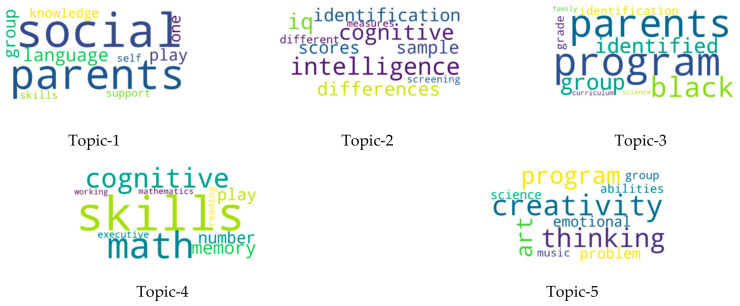
Topic-Based Word Clouds.

Figure 8 presents topic-based Word Cloud visualizations showing the intensity of the characteristic concepts associated with each theme identified by the BERTopic model. An examination of the visualizations indicates that each theme is concentrated around distinct keywords representing its conceptual focal points within the literature. In the first theme, concepts related to social interaction and family context are prominent, whereas the second theme is marked by a concentration of concepts associated with intelligence, IQ, and cognitive differences. In the third theme, concepts related to program access and identification processes become salient, while in the fourth theme, concepts concerning early academic skills come to the fore. In the fifth theme, concepts related to creativity and higher-order thinking skills are seen to carry greater weight.

**Figure 9:**
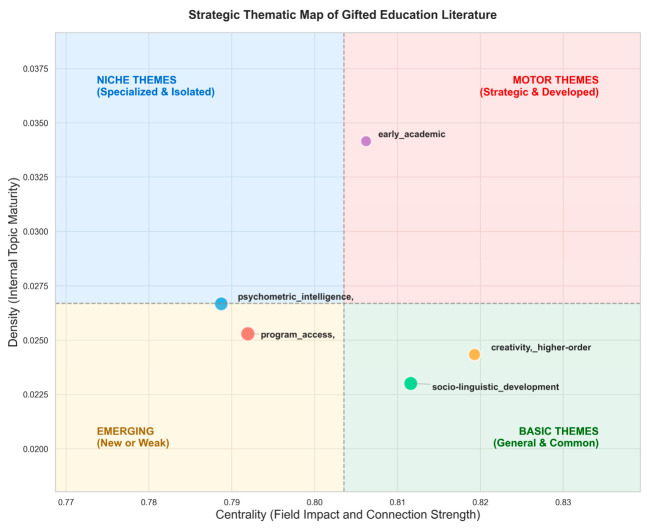
Thematic Map.

Figure 9 presents the strategic thematic map constructed to illustrate the positions of the themes identified by the BERTopic model within the field. The map was generated by positioning each theme on a two-dimensional plane according to its centrality and density values. Centrality refers to the strength of a theme’s connections with other themes and its level of inter-thematic interaction within the literature, whereas density indicates the conceptual coherence and research maturity of the theme itself. On the basis of these two measures, the themes are classified into four categories: motor themes (high centrality–high density), basic themes (high centrality–low density), niche themes (low centrality–high density), and emerging or declining themes (low centrality–low density).

The results indicate that the theme Early Academic Skills and Cognitive Development falls within the motor themes category, with high centrality and density values, and represents a well-developed and strategically important research focus in the literature. In contrast, Socio-Linguistic Development and Family Context and Creativity, Higher-Order Thinking, and Enrichment Programs are positioned in the basic themes category, with high centrality but relatively low density values, and represent foundational research areas with broad connections across the literature. On the other hand, Program Access, Identification, and Educational Equity and Psychometric Intelligence, Identification, and Cognitive Differences appear in the emerging themes area, with comparatively lower density and centrality values. This thematic structure indicates that some research areas constitute the core of the literature on giftedness in early childhood, whereas others are emerging as developing lines of inquiry.

**Figure 10:**
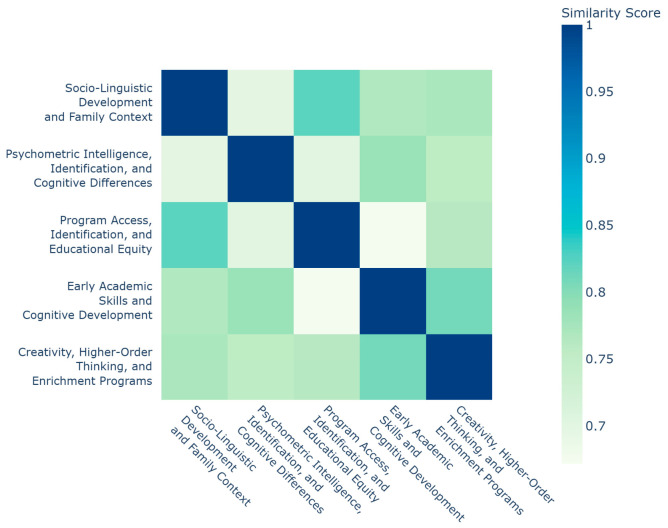
Topic Similarity Heatmap.

Figure 10 presents the topic similarity heatmap, which reveals the level of semantic proximity among the themes identified by the BERTopic model. The heatmap is based on the calculation of similarity scores between the document embedding representations associated with each theme and is intended to show the levels of conceptual overlap and differentiation among the themes. The values in the matrix indicate the degree of similarity between themes, with darker tones representing higher semantic proximity and lighter tones indicating relatively weaker conceptual relationships.

The similarity values obtained show that the themes are generally positioned around a moderate level of conceptual proximity, approximately within the range of 0.70 to 0.80. This indicates that the research areas within the literature on giftedness in early childhood do not form entirely independent thematic structures but are instead interconnected through certain conceptual points of intersection. In particular, the relatively high level of similarity between Early Academic Skills and Cognitive Development and Creativity, Higher-Order Thinking, and Enrichment Programs reflects the theoretical connections among cognitive development, higher-order thinking skills, and enrichment practices. By contrast, the comparatively lower similarity values observed between Program Access, Identification, and Educational Equity and the other themes suggest that this research area is positioned in the literature as a more specific and context-dependent area of discussion.

These findings demonstrate that the thematic structure generated by the BERTopic model not only produces distinct research clusters but also reveals the conceptual proximity and structural relationships among themes in the literature on giftedness in early childhood.

**Figure 11:**
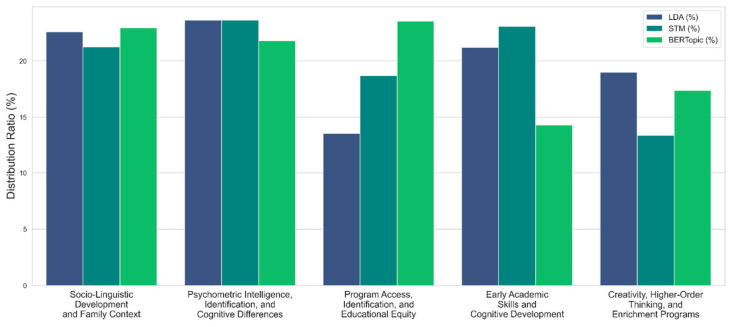
Subject Distribution Ratios.

Figure 11 comparatively presents the relative distribution ratios of the themes identified by the LDA, STM, and BERTopic models within the literature. The purpose of the figure is to show how different topic modeling approaches distribute the thematic structure across the same dataset and how the weight of particular research areas in the literature varies depending on the model.

The figure shows that in the LDA and STM models, the themes Psychometric Intelligence, Identification, and Cognitive Differences and Early Academic Skills and Cognitive Development are represented with relatively higher proportions. In the BERTopic model, by contrast, the theme, Program Access, Identification, and Educational Equity, is characterized by a higher distribution ratio. This suggests that the transformer-based semantic embedding approach is able to reveal semantic and policy-oriented research areas in the literature more distinctly.

In addition, the similar representation ratios of the theme Socio-Linguistic Development and Family Context across all three models indicate that this research area constitutes a stable thematic focus within the literature. By contrast, the theme Creativity, Higher-Order Thinking, and Enrichment Programs exhibits greater variation across the models, suggesting that the thematic representation of this research area may emerge differently depending on the modeling approach employed.

**Figure 12:**
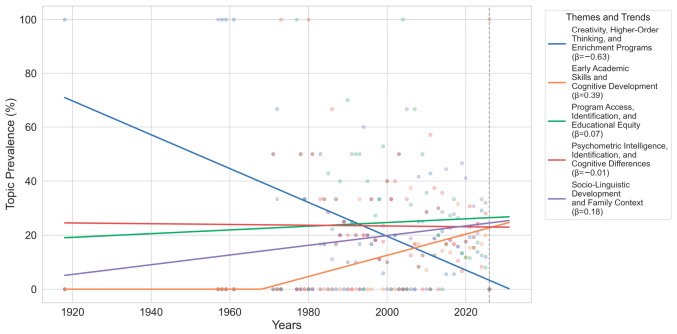
Temporal Trend Analysis of BERTopic Themes.

Figure 12 presents the results of the trend analysis conducted to reveal the direction and patterns of temporal change in the themes identified by the BERTopic model within the literature. The trend analysis was calculated using a linear regression model applied on the basis of each theme’s relative representation ratio in the literature (topic prevalence) across years. This approach aims not only to reveal the current thematic distribution but also to determine the directions in which the research field is developing and which themes may become more visible in the future. In the figure, the X-axis represents the years, while the Y-axis indicates the relative share of the themes in the literature (%). The slope coefficient (β) calculated for each theme represents the direction of increase or decrease over time.

The regression results indicate different trends across the themes. The positive slope coefficient for Socio-Linguistic Development and Family Context (β = 0.18) shows that studies focusing on social interaction and family processes have gained increasing importance in the literature. Similarly, the slight positive trend observed for Program Access, Identification, and Educational Equity (β = 0.07) suggests that access to educational programs and issues of equity have become an increasingly important research focus in recent years. By contrast, the marked negative slope coefficient for Creativity, Higher-Order Thinking, and Enrichment Programs (β = −0.63) indicates that the relative share of this research area in the literature has shown a declining trend over time. Early Academic Skills and Cognitive Development also exhibits a limited negative trend (β = −0.39). On the other hand, the slope coefficient of Psychometric Intelligence, Identification, and Cognitive Differences at β = −0.01 indicates that this research area has maintained a relatively stable thematic weight in the literature.

Taken together, these trends indicate that the literature on giftedness in early childhood has gradually shifted from approaches focused primarily on individual cognitive performance and academic skills toward research orientations that increasingly take into account environmental and educational factors such as social interaction, family dynamics, and equity in education. This pattern demonstrates that the intellectual structure of the field is not static but dynamic and evolving. By revealing these trends, the analysis makes it possible to identify emerging areas of research in the literature and thematic directions that may gain greater significance in the future.

## 4. Discussion

The study aimed to examine the conceptual structure of research on giftedness in early childhood, its thematic change over time, and the transformation of research trends through a comparison of LDA, STM, and BERTopic. The findings showed that this body of research is not grounded in a single understanding of giftedness. Rather, it reveals a multilayered and evolving structure in which psychometric, developmental, contextual, and pedagogical approaches coexist. In this respect, the findings related to the research questions were discussed in a multidimensional manner on the basis of international studies.

The findings related to the first research question indicate that the literature on giftedness in early childhood is shaped around five main thematic structures: (1) Socio-Linguistic Development and Family Context, (2) Psychometric Intelligence, Identification, and Cognitive Differences, (3) Program Access, Identification, and Educational Equity, (4) Early Academic Skills and Cognitive Development, and (5) Creativity, Higher-Order Thinking, and Enrichment Programs. This thematic diversity is strongly aligned with contemporary theoretical approaches that move beyond narrow and IQ test-oriented definitions of giftedness and instead conceptualize gifted potential as a contextual and multidimensional construct (Harrison, 2004; Hertzog, 2026; Lo & Porath, 2017; Walsh et al., 2012).

In terms of content, this thematic structure indicates that research on giftedness has moved away from a narrowly identification-centered focus, although it has not completely disengaged from this approach. The answer to the first research question is therefore not limited to simply listing themes. At the same time, these findings show that the conceptual framework surrounding giftedness has gradually expanded. This pattern is consistent with perspectives arguing that giftedness should be understood through multidimensional and context-sensitive approaches rather than through narrow definitions based solely on IQ (Rocha et al., 2026).

Particularly in early childhood, asynchronous development and the context-dependent emergence of potential make it difficult to address giftedness within a singular and fixed framework (Hernández-Torrano & Kuzhabekova, 2020). The prominence of socio-linguistic and family-oriented themes indicates that the literature increasingly approaches giftedness as a developmental characteristic that emerges through interaction, communication, and early environmental support. In early childhood, gifted potential manifests itself not primarily through tests, but through interaction, play, advanced vocabulary, and early environmental support mechanisms. This provides strong evidence that the field has begun to interpret giftedness not as a cognitive reservoir or merely an intelligence score, but as a complex and ecological condition (Hernández-Torrano & Kuzhabekova, 2020).

The second research question concerns how the BERTopic model makes thematic trends and shifts in orientation over time visible. The present findings show that this contribution is both methodological and substantive. From a methodological perspective, BERTopic was not selected on a hypothetical basis at the outset, but was preferred only after being compared with LDA and STM using the metrics of coherence, diversity, the Gini coefficient, and the silhouette score. The findings of the present study show that BERTopic provided comparatively clearer semantic differentiation, with a coherence value of 0.3958 and a positive silhouette score of 0.0468. This indicates that a clearer semantic differentiation was achieved across themes. Giftedness in early childhood is a conceptually heterogeneous field in which similar words may carry different meanings across domains such as cognition, assessment, curriculum, equity, and family processes. A frequency-based model may capture recurring word patterns, but a transformer-based semantic model can reveal contextual similarities in the literature more effectively. This finding is consistent with BERTopic’s architecture, which combines transformer embeddings, density-based clustering, and class-based TF-IDF (Galli et al., 2024), and with the way it differs from topic modeling approaches such as STM, which are sensitive to metadata but do not make use of contextual embeddings (Roberts et al., 2014).

More importantly, in this study, BERTopic made it possible to observe how the subject under investigation has changed and evolved over time. Trend analysis further reveals that the focal points of the literature have shifted over time. Socio-Linguistic Development and Family Context exhibited a positive slope (β = 0.18), while Program Access, Identification, and Educational Equity also showed an upward trend (β = 0.07). Psychometric Intelligence, Identification, and Cognitive Differences remained largely stable (β = −0.01), whereas Creativity, Higher-Order Thinking, and Enrichment Programs (β = −0.63) and Early Academic Skills and Cognitive Development (β = −0.39) declined relatively over time. These results do not indicate that certain themes have disappeared, but rather that the distribution of attention across the field has been rebalanced. Put differently, BERTopic makes visible how a literature once more strongly concentrated around cognitive performance and instructional concerns has gradually turned toward family processes, language development, and equity-sensitive access. This kind of thematic movement suggests that trends should be interpreted not merely as isolated statistics, but as indicators of broader epistemic transformations within the field (Şakar & Tan, 2025).

The findings of the present study show that identification continues to be a persistent and structurally strong area of research in the literature, while the surrounding field has expanded. This pattern strongly aligns with the view that, although gifted education has experienced paradigm shifts toward broader conceptions of talent development and inclusivity, the identification paradigm continues to persist implicitly (Lo & Porath, 2017). At the same time, it shows that identification remains one of the most contested and practically consequential areas in gifted education, because no single method can adequately capture the full range of gifted manifestations across different children (Kuznetsova et al., 2024; Marsili et al., 2025).

The third research question concerns the conceptual gaps that stand out in the literature and the areas that remain relatively underrepresented. The present findings indicate that, although the field has diversified, it has still matured unevenly. The visible rise of the theme Program Access, Identification, and Educational Equity in recent years is, in fact, a reflection of how historically neglected these issues have been within the field. The positive growth of family- and equity-oriented themes indicates that the visibility of these areas has increased. However, this rise itself also implies that these themes have historically not been given sufficient weight. In other words, these themes now appear as emerging areas precisely because they previously did not carry as much conceptual weight in the literature as psychometric identification, cognitive differences, and academic performance. The international literature has repeatedly shown that gifted programs are marked by significant inequalities in relation to race, language, socioeconomic status, and disability, and that traditional identification systems frequently render gifted potential invisible among underrepresented groups (Peters, 2022).

This issue may be even more pronounced in early childhood, because developmental expression is highly sensitive to variables such as language exposure, home learning conditions, opportunity structures, and adult interpretations. For this reason, the increasing visibility of the equity theme in the dataset of the present study reflects one of the most salient conceptual blind spots in the field, namely the unequal recognition of gifted potential across different sociocultural contexts. The fact that BERTopic renders this theme more visible than frequency-based models further suggests that semantically oriented topic modeling may be particularly useful for uncovering lines of research that are not dominant on the surface but carry important policy and justice implications.

The declining macro-trend exhibited by Creativity, Higher-Order Thinking, and Enrichment Programs (β = −0.63), one of the foundational themes of the field, requires careful analysis. Although creativity remains one of the five most dominant themes identified in this review, the marked negative trend suggests that this area has fallen behind other themes. This pattern, however, should not be interpreted as indicating that creativity has lost its importance. On the contrary, it may be argued that creativity-focused research in the literature on giftedness in early childhood has recently given way to more applied areas of emphasis. The most likely explanation for this trend is that creativity is no longer examined primarily as an independent object of research, but rather is increasingly studied in an implicit manner through its integration into contemporary domains such as early STEM, twenty-first-century skills, and neurocognitive research. This perspective is consistent with studies emphasizing the historical development of creativity (Naglieri & Kaufman, 2001; Sak, 2004), as well as with the recent literature indicating that higher-order thinking continues to retain its critical importance despite showing a heterogeneous distribution across publications (Lo & Feng, 2020). In addition, the continuing epistemological and methodological difficulties involved in the objective identification of creativity in early childhood may also have slowed the growth of this line of research.

The observed increase in Socio-Linguistic Development and Family Context (β = 0.18) reflects a significant paradigm shift in the conceptualization of early childhood giftedness. This trend suggests that researchers are increasingly directing their attention to the environments in which gifted potential first becomes visible. Unlike in later developmental periods, giftedness in early childhood often manifests through indicators such as linguistic complexity, advanced curiosity, symbolic play, memory, asynchronous development, or exceptionally developed social interaction. These indicators are typically first recognized by families and early childhood educators (Peyre et al., 2016).

The rise of this theme indicates that the field has begun to understand giftedness in early childhood through a more ecologically grounded perspective, giving greater consideration to the interpretive roles of parents, caregivers, and learning environments in the emergence and recognition of gifted potential.

The findings of this study offer a concrete vision for policymakers, educational administrators, and early childhood teachers. The scientific basis for evaluating gifted potential in early childhood solely through standardized tests, particularly traditional IQ tests, is becoming increasingly weak. In practice, dynamic and equitable identification mechanisms grounded in multiple data sources, including teachers’ pedagogical observations, portfolios, parent reports, and experience-based play-centered assessments, need to be integrated into the system. Otherwise, the risk that the potential of children from socioeconomically disadvantaged or culturally diverse groups will remain invisible becomes even more pronounced. In addition, the rise of socio-emotional and family-context themes highlighted by this study underscores that early intervention programs should systematically incorporate not only cognitive acceleration but also emotional support, parent guidance, and social adjustment.

The trend projections obtained suggest that future research on giftedness in early childhood will increasingly concentrate on themes that integrate developmental sensitivity with contextual sensitivity. While family context, socio-linguistic development, and equity-sensitive access are expected to gain further prominence, psychometric identification will likely persist not as a disappearing focus but as a stable yet central core of the field. This suggests that the next phase of the literature will focus less on whether giftedness in early childhood can be recognized and more on how it is recognized, by whom it is recognized, under what conditions it becomes visible, and what consequences this has for inclusivity and educational opportunity. Such a direction closely aligns with current critiques calling for greater conceptual clarity in socio-emotional and contextual research, as well as for more equitable and multidimensional identification practices (Rinn, 2024). At the same time, the relative decline in the visibility of creativity and early academic skills suggests that these areas should also be theoretically and empirically reinforced so that the field does not become overly concentrated on access and identification alone.

## 5. Conclusions

This study examined the literature on giftedness in early childhood through 518 peer-reviewed articles compiled from the Scopus and Web of Science databases and comparatively evaluated the LDA, STM, and BERTopic models on the same dataset. The findings indicate that the BERTopic model provides a stronger framework at both the methodological and substantive levels. In particular, a topic coherence value of 0.3958 showed that BERTopic outperformed STM by approximately 11% and LDA by approximately 34% in terms of within-topic conceptual coherence. Similarly, a diversity value of 0.8200 indicated lower overlap across topics and more distinct thematic differentiation. In addition, the low Gini coefficient value of 0.0396 demonstrated a more balanced distribution of themes across the dataset, while the positive silhouette score (0.0468) showed that BERTopic was able to distinguish documents more clearly than the other models in semantic embedding space. Taken together, these findings suggest that BERTopic is a more suitable model not only statistically, but also in terms of semantic interpretability, thematic structuring, and conceptual differentiation.

This research goes beyond merely describing the literature at the level of topic labels and instead presents a holistic temporal and conceptual mapping of the field. In this respect, the comparison of analytical methods, the identification of thematic distribution patterns, and the use of thematic mapping, topic similarity analyses, and trend analyses made it possible to render visible the proximities, distinctions, and directions of change among themes over time. Accordingly, the study demonstrates both how methodological choices are reflected in the conceptual representation of the literature and around which thematic axes the field is concentrated, as well as how these axes have been reshaped over time.

BERTopic-based content analyses showed that the literature is structured around five main thematic axes. These axes consist of Socio-Linguistic Development and Family Context, Psychometric Intelligence, Identification, and Cognitive Differences, Program Access, Identification, and Educational Equity, Early Academic Skills and Cognitive Development, and Creativity, Higher-Order Thinking, and Enrichment Programs. The trend results revealed that studies focused particularly on family context, socio-linguistic development, and equity have shown a rising trajectory, whereas psychometric identification has remained a central yet more stable axis of the field. These findings suggest that, within the analyzed dataset, research on giftedness in early childhood shows a relative movement from narrowly identification-centered approaches toward themes related to family context, socio-linguistic development, and educational equity. Therefore, the study provides a corpus-based basis for identifying thematic areas that may warrant closer attention in future research.

## 6. Limitations and Recommendations

The findings of this study should be interpreted within the framework of certain methodological limitations. First, the final analytic sample consisted of 518 peer-reviewed journal articles retrieved from Scopus and Web of Science. Although this corpus provides a systematic basis for topic modeling, its size and database scope limit the extent to which the findings can be generalized to the entire field. Second, the analyses were conducted on article abstracts, and the detailed contextual structure that full texts could provide was not captured. Because the study is based on bibliographic records and abstracts rather than participant-level primary data, variables such as participant demographics, prior educational experience, socioeconomic background, and institutional context could not be directly controlled. Therefore, the findings should be interpreted as corpus-level thematic trends rather than causal or participant-level inferences. Third, the sensitivity of the HDBSCAN parameters used in the BERTopic model may lead to partially different thematic structures under alternative parameter settings. Finally, the linear regression approach used in the trend analysis may not fully capture the nonlinear dimensions of thematic change.

Future research may benefit from the use of full-text analyses to provide a more comprehensive mapping of the field. In addition, supporting computational analyses with qualitative and mixed-method approaches may help reveal more nuanced thematic patterns in the literature.

## Figures and Tables

**Table 1:** Search Dataset Information.

Source	Search	Valid
Scopus	424	389
WoS	321	310
Total	745	699
Duplicates	181	
Total Valid (*n*)	518	

**Table 2:** Keywords Extracted According to Models.

Topics	BERTopic	STM	LDA
Topic-1	social, parents, language, group, play	program, skills, social, group, problem	social, effect, ability, cognitive, practice
Topic-2	intelligence, iq, cognitive, differences, identification	group, language, cognitive, abilities, low	skill, social, group, difference, effect
Topic-3	program, parents, black, group, identified	skills, play, parents, social, role	language, group, state, artistic, showed
Topic-4	skills, math, cognitive, memory, play	parents, differences, reading, emotional, skills	parent, cognitive, program, science, emotional
Topic-5	creativity, thinking, program, art, problem	creativity, thinking, black, one, work	identification, group, identified, model, parent

**Table 3:** Comparison of Metrics by Model.

Metrics	BERTopic	STM	LDA
Coherence	0.3958	0.3554	0.2958
Diversity	0.8200	0.7000	0.7200
Gini	0.0396	0.0997	0.0952
Silhouette	0.0468	−0.0236	−0.0461

**Table 4:** BERTopic and emerging academic themes.

Topics	BERTopic Keywords	Thema
Topic-1	social, parents, language, group, play	Socio-Linguistic Development and Family Context
Topic-2	intelligence, iq, cognitive, differences, identification	Psychometric Intelligence, Identification, and Cognitive Differences
Topic-3	program, parents, black, group, identified	Program Access, Identification, and Educational Equity
Topic-4	skills, math, cognitive, memory, play	Early Academic Skills and Cognitive Development
Topic-5	creativity, thinking, program, art, problem	Creativity, Higher-Order Thinking, and Enrichment Programs

## Data Availability

The data analyzed in this study were obtained from Scopus and Web of Science and are available from the corresponding author upon reasonable request, subject to the terms and conditions of these databases.
